# Src1 is a Protein of the Inner Nuclear Membrane Interacting with the *Dictyostelium* Lamin NE81

**DOI:** 10.3390/cells5010013

**Published:** 2016-03-18

**Authors:** Petros Batsios, Xiang Ren, Otto Baumann, Denis A. Larochelle, Ralph Gräf

**Affiliations:** 1 Institut für Biochemie und Biology, Department of Cell Biology, Universität Potsdam, Karl-Liebknecht-Str. 24-25, 14476 Potsdam, Germany; batsios@uni-potsdam.de; 2 Department of Biology, Clark University, 15 Maywood St., Worcester, MA 01610-1477, USA; xren@clarku.edu (X.R.); dlarochelle@clarku.edu (D.A.L.); 3 Institut für Biochemie und Biology, Department of Animal Physiology, Universität Potsdam, Karl-Liebknecht-Str. 24-25, 14476 Potsdam, Germany; obaumann@uni-potsdam.de

**Keywords:** *Dictyostelium*, lamin, nuclear lamina, nucleus, nucleolus, HeH-protein, LEM-domain protein

## Abstract

The nuclear envelope (NE) consists of the outer and inner nuclear membrane (INM), whereby the latter is bound to the nuclear lamina. Src1 is a *Dictyostelium* homologue of the helix-extension-helix family of proteins, which also includes the human lamin-binding protein MAN1. Both endogenous Src1 and GFP-Src1 are localized to the NE during the entire cell cycle. Immuno-electron microscopy and light microscopy after differential detergent treatment indicated that Src1 resides in the INM. FRAP experiments with GFP-Src1 cells suggested that at least a fraction of the protein could be stably engaged in forming the nuclear lamina together with the *Dictyostelium* lamin NE81. Both a BioID proximity assay and mis-localization of soluble, truncated mRFP-Src1 at cytosolic clusters consisting of an intentionally mis-localized mutant of GFP-NE81 confirmed an interaction of Src1 and NE81. Expression GFP-Src1^1–646^, a fragment C-terminally truncated after the first transmembrane domain, disrupted interaction of nuclear membranes with the nuclear lamina, as cells formed protrusions of the NE that were dependent on cytoskeletal pulling forces. Protrusions were dependent on intact microtubules but not actin filaments. Our results indicate that Src1 is required for integrity of the NE and highlight *Dictyostelium* as a promising model for the evolution of nuclear architecture.

## 1. Introduction

In animal cells, the nuclear envelope is composed of an outer nuclear membrane (ONM), which is continuous with the endoplasmic reticulum, and an inner nuclear membrane (INM) supported by the nuclear lamina [1]. The inner and outer nuclear membrane are separated by the perinuclear space and contiguous with each other at nuclear pore complexes, which are also in contact with the nuclear lamina. The latter refers to a protein network consisting of inner nuclear membrane proteins and a network of type V intermediate filaments called lamins. Lamins are capable of forming flexible protofilaments that may assemble to higher order structures beneath the INM. Yet, how protofilaments are organized *in vivo* and in different species remains uncertain [2,3]. There are two types of lamins, A-type and B-type. While B-type lamins are expressed in all cells, A-type lamins are present only upon differentiation. Lamin A and lamin B proteins are expressed as pre-proteins with a C-terminal CaaX-box that serves as a prenylation site for anchorage to the INM. In A-type lamins the prenyl group together with the last 15 amino acids is cleaved off prior to filament assembly, while it persists in B-type lamins. A- and B-type lamin networks interact directly or indirectly with more than 80 different proteins, many of which are transmembrane proteins of the INM [4]. These include Sun-proteins linking the lamin network through the nuclear envelope to the cytosolic cytoskeleton via so-called LINC complexes [5] and proteins of the helix-extension-helix (HeH) superfamily of DNA-binding INM proteins [6]. Among the latter is a group of intensively-studied proteins known as LEM-domain proteins, named for a shared, conserved domain found in lamina-associated polypeptide 2 (LAP2), Emerin, and MAN1 [7]. In metazoans, the LEM-domain associates with the nucleoplasmic chromatin linker protein BAF (barrier to autointegration factor) and, thus, provides one means to tether portions of chromatin to the nuclear lamina [8]. LAP2 isoforms additionally contain a related LEM-like domain that is capable of binding to double stranded DNA directly [9]. Various studies have shown that chromatin-lamina interactions are crucial in gene regulation, especially epigenetic gene silencing by heterochromatin formation in the nuclear periphery [10]. LEM-domain proteins fall into three groups, one with family members containing one transmembrane domain (I), one with two transmembrane domains (II), and one lacking transmembrane domains but containing ankyrin-repeats (III) [6]. Unicellular eukaryotes also express inner nuclear membrane proteins related to LEM-proteins. The first of these proteins to be identified was budding yeast, Src1p (alternative name Heh1p), whose mutation caused accelerated sister chromatid segregation [11]. Later results suggested a major role of Src1 in nucleolar organization. The main function of Src1p appears to lie in stabilization of the highly-repetitive rDNA sequences at the periphery of budding yeast nuclei [12]. Its orthologue in *Schizosaccharomyces japonicus*, Man1, appears to be required for nucleolar disassembly [13]. In all of these fungal proteins the canonical LEM motif is replaced by a similar helix-extension-helix (HeH) domain that can interact with DNA directly, possibly due to the lack of BAF in unicellular eukaryotes [14]. This suggests that the LEM-domain has co-evolved from the HeH-domain together with the emergence of BAF [6]. All LEM-domain family members in non-metazoan organisms belong to group II with two membrane domains and the N- and C-termini facing the nucleoplasm. Group II proteins also contain a second, conserved MSC-domain (MAN1/Src1p/C-terminal) at their C-terminus. In humans the MSC-domain of MAN1 forms a winged helix fold that is capable of interacting with DNA directly [15]. 

In lower eukaryotes little is known about the relationship of MAN1-like proteins to the nuclear lamina. This is because among lower eukaryotes nuclear lamina proteins evolutionarily related to lamins have so far been characterized only in the amoebozoan *Dictyostelium discoideum* [16]. With regard to its primary structure and all experimental results, the coiled-coil protein NE81 meets all requirements of a *bona fide* lamin. It is associated with the INM requiring a CaaX-box for prenylation to do so. Furthermore, it appears to be capable of CDK1-dependent assembly/disassembly, is required for mechanical integrity of the cell, and mediates linkage of the centrosome to the nucleus [17,18]. Among the INM proteins, we have recently shown by proximity-dependent biotin identification (BioID) that NE81 also displays the conserved interaction of Sun1 with lamins [19]. The discovery of NE81 in *Dictyostelium* and, most recently, identification of putative orthologues also in the SAR group of organisms (Stramenopile, Alveolata, Rhizaria) [20] indicates that the last common ancestor of eukaryotes (LECA) already possessed lamins in addition to HeH-proteins and Sun-proteins [21,22]. In this paper we provide the first characterization of a MAN1-like HeH-family protein, Src1, in an amoebozoan, and show by light and electron microscopy that Src1 is an INM protein that interacts with the *Dictyostelium* lamin NE81 in BioID and mis-localization assays. These findings corroborate the value of *Dicytostelium* as a model to study basic functions of nuclear envelope organization, since among all other model organisms it appears to reflect the situation in LECA most closely.

## 2. Materials and Methods 

### 2.1. Vector Constructions and Expression of Recombinant Src1 for Immunizations 

To generate the GFP-Src1 construct, genomic DNA was used as a template for PCR amplification of the complete Src1 sequence from the initiator ATG to the stop codon using SalI-forward and BamHI-reverse linker primers. The PCR product was cloned into the N-terminal GFP-fusion vector pIS76 [23] to yield pPB130 (blasticidin resistance). All further Src1 constructs are based on this plasmid. BirA and BirA-NE81 strains were generated as described previously [19] and used for BioID as described [19]. pPB130 was used as a PCR template to generate the mRFP-Src1^356–565^ and mRFP-Src1^826–942^ truncation constructs using appropriate SalI-forward and BamHI-reverse linker primers (numbers refer to the amino acid sequence). These fragments were cloned into the pIS254 vector (pIS76, in which GFP was replaced by marsRFP [24]) to yield plasmids pPB134 (blasticidin) and pPB135 (blasticidin), respectively. To generate the GFP-NE81ΔNLSΔCLIM vector, pAK35 [18] was used as a template for PCR amplification of two PCR products overlapping in the NLS encoding region, which was mutated in the respective primers in order to replace all basic NLS residues by alanine residues. Primer combinations were as follows: left PCR fragment VP3-1 SalI: TAAATTGTCGACTAATGGATATGTCAAAAAAGAAAAGTAAAC, NE81_Ala_NLS_R: TGCTGCTGCCGCTGCCGCAGCATCATCAACGGTCTTTTCAAAACCT; right PCR fragment: NE81_Ala_NLS_F: GCTGCGGCAGCGGCAGCAGCACTTCAACATGAATTCAATGCTGCTG, RNE81_BamHI-CLIM: GCGCGGATCCTTAATTTGATTTACCAGCTGAAGAAGG. The generated PCR products were annealed at the mutated overlapping NLS regions and used for a third overlap extension PCR using the oligonucleotides VP3-1 SalI and RNE81_BamHI-CLIM. The final PCR product with the inactivated NLS and deleted C-terminal CaaX motif was cloned into the N-terminal GFP-fusion vector pIS77 [23] to yield pPB96 (G418 resistance). To generate the GFP-Src1 (1_646) vector, genomic DNA was used as a template for PCR amplification of the DNA sequence encoding the 2nd (ATG is omitted to avoid internal translation) to the 646th amino acids of Src1 protein. The PCR product was cloned into the N-terminal GFP-fusion vector pDM351 (G418 resistance) [25] by Gateway cloning (Thermo Fisher Scientific, Braunschweig, Germany).

*Dictyostelium* cells (strain AX2) were grown in HL5c medium (Formedium, Hunsanton, UK) at 21 °C either adherent in tissue culture flasks or in suspension in Erlenmeyer flasks on a rotary shaker at 150 rpm. Cells were transformed by electroporation as described earlier [26].

For expression of Src1 in *Escherichia coli*, the C-terminal sequence encoding amino acids 826–942 was amplified by PCR as described above and was cloned into a modified version of pMALc2 (NEB, Frankfurt, Germany), in which the original polylinker was replaced by a SalI, PstI, EcoRI, BamHI, HindIII linker (pIS248). Protein expression at room temperature and purification by amylose affinity chromatography was performed according to the manufacturer's instructions. The fusion protein was used for custom immunization of two rats (Preclinics, Potsdam, Germany). Antisera were used directly for IF and Western blot analysis.

### 2.2. Microscopy

Light microscopy and image processing of fixed samples (whole cells or isolated nuclei prepared according to [27]) were conducted as described previously on a Zeiss CellObserver HS system (Carl Zeiss, Jena, Germany) equipped with a PlanApo 1.4/100× objective, an Axiocam MRm Rev. 3 CCD camera, a piezo stage and the Axiovision 4.7 iterative deconvolution software package [23]. Maximum intensity projections of deconvolved image stacks (focus step size 0.25 μm) were calculated with Axiovision. Mean staining intensities at the nuclear envelope were measured with ImageJ. Therefore the background was set to zero and regions at the nuclear envelope with and without attached nucleoli (nucleoli were localized by their typical dark appearance in phase contrast images) were selected using the freehand selection tool. For normalization, the mean staining intensity at the nucleolar region was set to 1. Live cells were prepared in glass-bottom Petri dishes (Fluorodish, WPI, Berlin, Germany) and flattened by an agar overlay [28]. Live cell imaging was performed as described recently [29] on a spinning disc confocal microscope (CellObserver SD, Carl Zeiss, Jena, Germany) equipped with a PlanApo 1.4/100x objective, two Evolve EM-CCD cameras (Photometrics, Tucson, AZ, USA), and a Rapp UGA-40-2L Galvo Scanner (Rapp Optoelectronics, Hamburg, Germany) for laser manipulations. Image stacks consisting were recorded as indicated in the movie legends. For FRAP experiments a square region of interest covering less than 10% of the area of the cell was bleached with the 473 nm laser line of the Rapp UGA-40-2L system at maximum laser intensity. Fluorescence intensities during recovery of the moving region of interest were measured with ImageJ within a 4 × 4 square pixel area (0.251 μm^2^). FRAP experiments were evaluated according to [30].

Electron microscopy of isolated nuclei was performed as published recently [27]. For immuno-EM, nuclei were fixed with glutaraldehyde, labeled with anti-GFP and nanogold-conjugated anti-rabbit Fab' fragments (Aurion, Wageningen, The Netherlands), silver enhanced, osmicated, and finally embedded in Spurr's resin. Uranyl acetate and lead citrate stained ultra-thin sections were viewed on a Philips CM100 electron microscope.

### 2.3. Membrane Protein Extractions, Electrophoresis, and Western Blotting

GFP-Src1-646 cells were used for membrane protein extraction experiments. Total cell lysates were obtained by filtration through 5 μm Nucleopore track-etched membranes (Whatman GmbH, Dassel, Germany). Intact nuclei were collected by centrifugation at 4000 rpm (Sorvall SH-3000 rotor) for 15 min at 4 °C. The intact nuclei were incubated with IPP150 buffer (10 mM Tris–HCl (pH 8.0), 150 mM NaCl, 0.1% NP-40), IPP150 buffer + 1% Triton or IPP150 buffer + 1 M NaCl) at 4 °C for 30 min. After incubation, pellet and supernatant were separated by microcentrifugation at maximum speed for 20 min at 4 °C. SDS electrophoresis and Western blotting was carried out as described [31]. Densitometric measurements of immunoblot bands and fluorescence images were undertaken with the ImageJ program.

### 2.4. Antibodies and Streptavidin Conjugates

Primary antibodies: rat anti-Src1 (this work), rabbit anti-NE81 [18], rat YL1/2 [32], rabbit anti-GFP [33], rabbit anti-GFP (Molecular Probes, A-6455; Life Technologies, Carlsbad, CA, USA). AlexaFluor conjugated secondary antibodies and streptavidin-AlexaFluor 488 were purchased from Life Technologies (Carlsbad, CA, USA), streptavidin-CIP (calf intestine alkaline phosphatase) and anti-rabbit-CIP/anti-rat-CIP from Sigma-Aldrich (Deisenhofen, Germany).

## 3. Results

**Figure 1:**
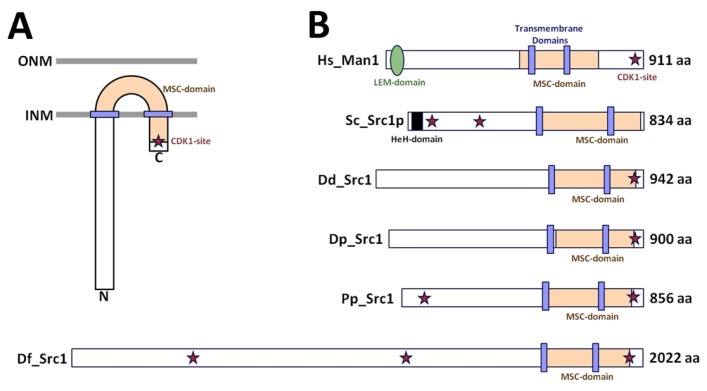
Domain organization of class II LEM-domain proteins. (**A**) Experimentally-confirmed orientation of class II LEM-domain proteins. (**B**) Schematic alignment of human MAN1 (*H.sapiens*, Hs_Man1, Q9Y2U8) with HeH-family proteins in budding yeast (*S. cerevisiae*, Sc_Src1p, AJS93713), and Dictyostelidae (*D. discoideum*, Dd_Src1, DDB0306789; *D. purpureum*, Dp_Src1, DPU1260054; *P. pallidum*, Pp_Src1, PPA1422192; *D. fasciculatum*, Df_Src1, DFA1446660). Relative positions of the LEM-domain, HeH-domain, MSC-domains, transmembrane domains and predicted CDK1 phosphorylation (asterisk) are indicated.

The *Dictyostelium discoideum* genome encodes a single gene product belonging to the HeH-protein family. It is annotated as *src1*, since it represents the closest homologue to yeast Src1p [34]. *Dictyostelium* Src1 is a 942 amino acid protein with a calculated molecular mass of 107 kDa. It contains two predicted transmembrane sequences (609–631 and 807–826) and an MSC-domain characteristic of HeH-proteins (624–914). Obvious amino acid sequence similarity to proteins in non-dictyostelid species is restricted to the MSC-domain with an amino acid sequence identity/similarity to *S. cerevisiae* Src1p of 23%/35%, respectively. Unlike other proteins of this family *Dictyostelium* Src1 contains no HeH-domain. This property is shared by the Src1 orthologues in other *Dictyostelidae*, which show only low overall sequence identity except for the MSC domain (MSC domain identities: *D. purpureum* 65%, *D. fasciculatum* 39%, *Acytostelium subglobosum* 38%, and *Polysphondylium pallidum* 33%). Yet, domain structures are similar in all dictyostelid orthologues, except for the large N-terminal extension in *D. fasciculatum* Src1, whose sequence is more than twice as long as all other Src1 orthologues (Figure 1).

**Figure 2:**
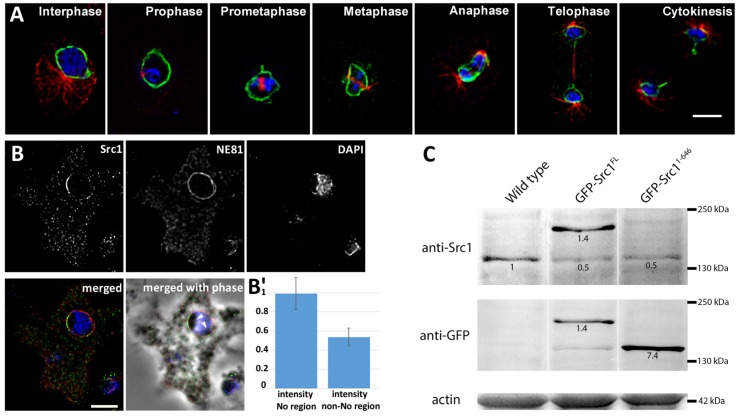
Distribution of Src1 at the nuclear envelope. (**A**) Localization of GFP-Src1 during interphase and mitosis. Mitotic stages are indicated. GFP fluorescence is shown in green, DAPI staining of nuclei in blue, and microtubules in red (labeled with anti-α-tubulin YL1/2 and anti-rat-AlexaFluor 561). Bar, 4 μm. (**B**) Localization of endogenous Src1 labeled with anti-Src1/anti-rabbit AlexaFluor 488 (green), anti-NE81/anti-AlexaFluor 568 (red), and DAPI (blue). Note the uneven distribution of Src1 with accumulations at sites of nucleolar attachment to the nuclear envelope. Nucleoli (arrowheads) are poorly stained with DAPI and visible in phase contrast images as dark structures within the nucleus. (**A, B**) widefield deconvolution microscopy. Specimens were fixed with glutaraldehyde. Maximum intensity projections of thin image stacks through the center of the nucleus are shown. Bar, 5 μm. (**B'**) Quantitative evaluation of anti-Src1 staining intensities in nucleolar (No) *vs.* non-nucleolar (non-No) regions at the NE. Mean normalized staining intensities ± S.D. are shown (*n* = 60 for each region). (**C**) Western blot of cytosolic extracts of wild-type, GFP-Src1 full length, and GFP-Src1^1–646^ cells stained with anti-Src1 antibodies and anti-GFP antibodies as indicated. Numbers refer to relative staining intensities, whereby Src1 staining in wild-type cells was set to 1. As protein levels of GFP-Src1 are the same whether stained with anti-Src1 or anti-GFP, the protein level of GFP- Src1^1–646^ compared to endogenous Src1 could be deduced. Actin is shown as a loading control.

To confirm that Src1 is indeed a protein residing in the nuclear membrane(s) we have generated a *Dictyostelium* strain expressing Src1 fused to GFP at its N-terminus. GFP-Src1 is associated with the nuclear envelope during interphase and mitosis. During mitosis the nuclear envelope stays intact until it ruptures upon karyokinesis in the late telophase (Figure 2A, supplemental movie 1) [35,36]. Nuclear envelope association of Src1 was confirmed in immunofluorescence specimens stained with antibodies raised against a C-terminal, soluble Src1 fragment (aa 826–943), which clearly showed Src1 co-localization with NE81 at the nuclear envelope. However, while NE81 was evenly distributed along the whole nuclear envelope, staining against Src1 was patchy with increased staining intensities at sites where the nuclear envelope contacts nucleoli (Figure 2B).

In *Dictyostelium* multiple nucleoli (typically two to four) are always found at the nuclear periphery and characterized by low staining with DAPI [37,38,39]. Quantitative evaluation revealed that anti-Src1 staining at the NE was ~1.85-fold stronger in regions associated with nucleoli (Figure 2B'). The bias of Src1 staining toward nucleolar regions was much less pronounced in case of GFP-Src1 (Figure 2A), which is most likely due to overexpression of the fusion protein (see Figure 2C). 

**Figure 3:**
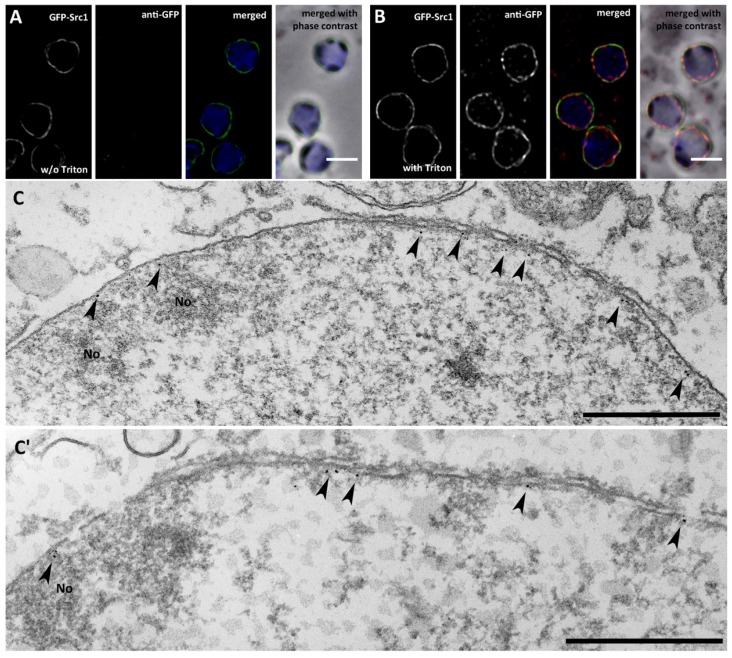
Src1 is an inner nuclear membrane protein. (**A, B**) Isolated nuclei of GFP-Src1 cells were stained with anti-GFP-antibodies [33] either in the absence (**A**) or presence (**B**) of 0.5% Triton-X100. The antibody is accessible to GFP-Src1 only upon permeabilization of the nuclear membranes. Specimens were fixed with glutaraldehyde. Bar, 2 μm. (**C, C'**) Two examples of immuno-transmission electron microscopy of isolated nuclei stained with anti-GFP/anti-rabbit nanogold. Gold particles (arrowheads) are visible only along the inner nuclear membrane. Bar, 500 nm.

Next, we tested whether Src1 is an exclusive INM protein, and whether the N-terminus of this two transmembrane helix protein faces the nucleoplasm, as true for other Man1 orthologues [7,40], or projects into the perinuclear space. GFP-Src1 was stained with anti-GFP antibodies only if isolated nuclei were first permeabilized with Triton X-100, suggesting that GFP-Src1 is targeted exclusively to the inner nuclear membrane (Figure 3A,B). Although attempts to stain endogenous Src1 with our anti-Src1 antibodies in immuno-electron microscopy specimens failed, labeling of GFP-Src1 with anti-GFP antibodies decorated with gold-labeled F_ab_-fragments worked well. Gold particles were solely detected on the nucleoplasmic side of the INM indicating that the Src1 N-terminus is oriented towards the nucleoplasm (Figure 3C). The existence of two predicted transmembrane domains implies that this holds true also for the C-terminus. Taken together, we present strong evidence that Src1 is an inner nuclear membrane protein with both its amino- and carboxy-termini oriented towards the nucleoplasm. In agreement with GFP-Src1 distribution in fluorescence microscopy we found no bias in the distribution of gold particles at the NE towards nucleoli (Figure 3C,C').

**Figure 4:**
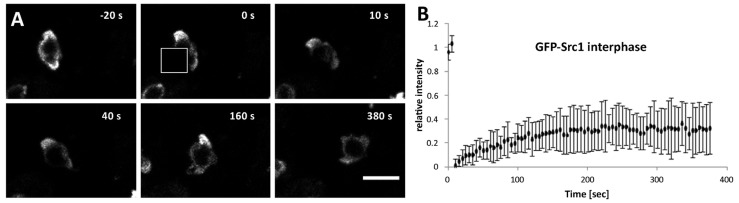
GFP-Src1 exhibits low mobility in FRAP experiments. (**A**) Selected time points of supplemental movie 2 are shown. The bleached region is indicated by a white square. Bar, 5 μm. (**B**) FRAP curve showing mean values ± S.D. after normalization and background correction (*n* = 8).

To assess whether Src1 could be a component of the nuclear lamina we employed fluorescence recovery after photobleaching (FRAP) of nuclear envelopes of GFP-Src1 cells (Figure 4, supplemental movie 2). The overall low recovery of GFP-Src1 after photobleaching supports the hypothesis that Src1 is part of the nuclear lamina. However, standard deviation values of recovery curves were relatively high indicating that GFP-Src1 may possess different recovery kinetics at different regions of the nuclear envelope, like for example at nucleolar *versus* non-nucleolar regions. Unfortunately, we were unable to investigate whether GFP-Src1 recovery kinetics were different at nucleoli and non-nucleolar regions of the NE, as the diffraction limited bleaching spot was too large to clearly distinguish between these regions.

**Figure 5:**
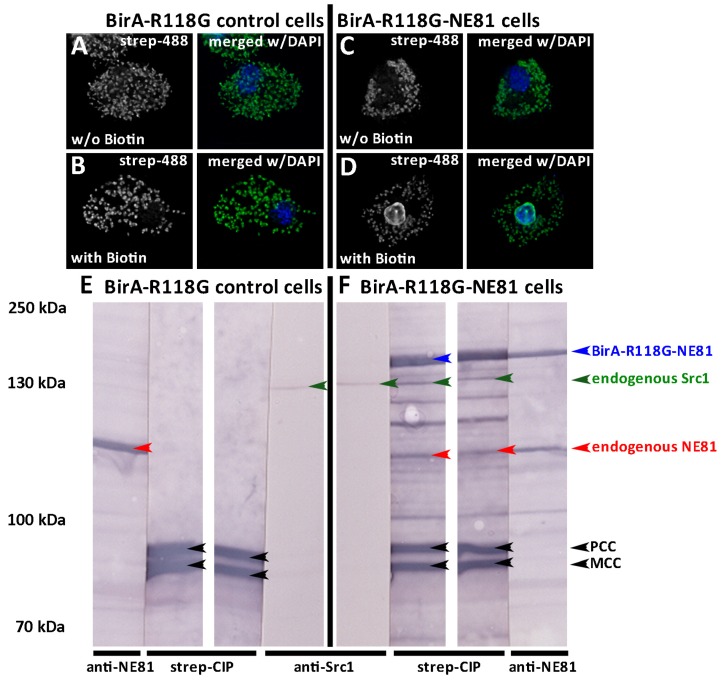
BirA-R118G-NE81 biotinylates itself and Src1. (**A-D**) Fluorescence microscopy of glutaraldehyde fixed BirA-R118G control cells (**A, B**) and BirA-R118G-NE81 cells (**C, D**) stained with streptavidin-AlexaFluor 488 (green) and DAPI (blue). Cells were cultivated with (**B, D**) or without (**A, C**) addition of biotin to the medium as indicated. Note the biotin-labeling of the nuclear envelope in (**D**). Mitochondria are stained as well due to the presence of constitutively biotinylated proteins [19]. Bar, 5 μm. (**E, F**) Western blot of nuclear extracts from BirA-R118G control cells. Positions of marker proteins are given on the left. (**E**) and BirA-R118G-NE81 cells (**F**) stained with anti-NE81/anti-rabbit-CIP, streptavidin-CIP and anti-Src1/anti-rabbit-CIP. Color detection was performed with nitroblue tetrazolium chloride (NBT) and bromo-chloro-indolyl-phosphate (BCIP). Blot lanes were split in two halves, stained individually as indicated and re-aligned after staining as shown. Positions of BirA-R118G-NE81 (blue arrowhead), Src1 (green arrowhead) and NE81 (red arrowhead) are shown. Black arrowheads indicate positions of known constitutively biotinylated mitochondrial proteins [19]. Src1 and NE81 are only biotinylated in BirA-R118G-NE81 cells, but not in control cells. BirA-NE81 also biotinylates itself [19].

According to our recent analyses, the lamin NE81 is the major component of the nuclear lamina in *Dictyostelium*. Thus, we suspected an interaction of Src1 with NE81 and pursued two independent experimental strategies to test this hypothesis. First, we used the BioID (biotin identification) proximity assay, in which a protein of interest is tagged with a mutated variant of the *E. coli* biotinylase BirA (BirA-R118G) to promiscuously biotinylate all binding partners (and itself) within a proximity of approximately 10 nm or less [41,42]. Thus, BioID operates in the same proximity range as FRET analyses. Recently we have adapted this method for use in *Dictyostelium* amoebae and have successfully demonstrated that BirA-R118G-tagged NE81 biotinylates Sun1 *in vivo*, showing that the established interaction between lamins and the Sun1 INM protein family is also conserved in amoebae [19]. Here we used the same BirA-R118G-NE81 strain to show specific, proximity-dependent biotinylation of Src1. Fluorescence microscopy of BirA-R118G-NE81 cells with AlexaFluor-488-conjugated steptavidin confirmed specific biotinylation of nuclear envelope proteins (Figure 5A–D). Western blots with nuclear protein extracts from BirA-R118G control cells and BirA-R118G-NE81 cells were stained either with a streptavidin-phosphatase conjugate or specific antibodies against NE81 and Src1 (Figure 5E,F). To facilitate comparisons of individual stainings, Ponceau S-stained blot lanes were cut in the middle so that both halves of the lane could be used for probing with two different antibodies, respectively, and subsequently aligned perfectly aside each other. Staining with anti-NE81 shows biotinylation of endogenous NE81 and BirA-R118G-NE81 as reported earlier [19]. Staining with anti-Src1 clearly revealed a band that is positive for streptavidin staining and absent in control cells expressing only BirA-R118G, strongly indicating an interaction between Src1 and NE81 (Figure 5E,F).

**Figure 6:**
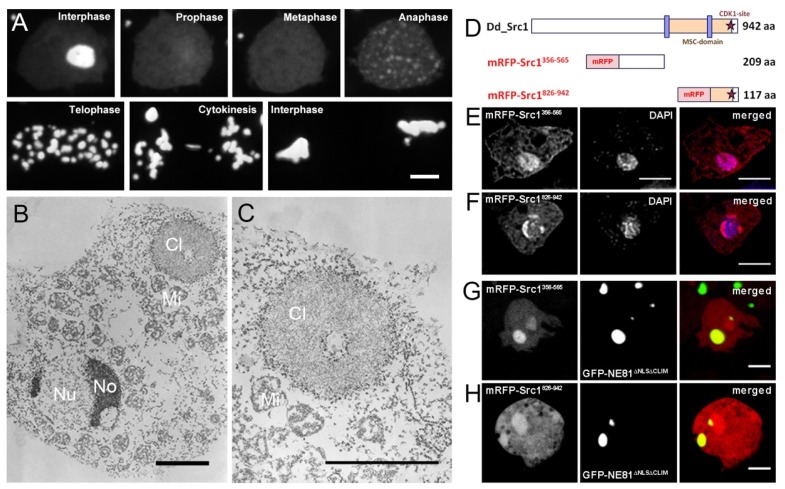
mRFP-Src1^356–565^ and mRFP-Src1^826–942^ localize to cytosolic GFP-NE81ΔNLSΔCLIM clusters. (**A**) Selected time points of supplemental movie 3 showing the dynamic behavior of GFP-NE81ΔNLSΔCLIM clusters. (**B, C**) Transmission electron microscopy showing spongy GFP-NE81ΔNLSΔCLIM clusters (Cl) studded by particles representing ribosomes. The nucleus (Nu), nucleoli (No) and mitochondria (Mi) are labeled. (**C**) is an enlarged view of (**B**). (**D**) Schematic of mRFP-Src1 fragments used in (**E–H**). (**E, F**) mRFP-Src1^356–565^ and mRFP-Src1^826–942^ (red) predominantly localize to the nucleus (stained with DAPI, blue). mRFP-Src1^826–942^ is concentrated at regions with low DAPI staining indicating presence at the nucleoli. Cells were fixed with glutaraldehyde. (**G, H**) In live GFP-NE81ΔNLSΔCLIM cells mRFP-Src1^356–565^ and mRFP-Src1^826–942^ (red) mainly localize to GFP-NE81ΔNLSΔCLIM clusters. Bars, 5 μm.

To independently prove the Src1-NE81 interaction we tested for mis-localization of Src1 to artificial, cytosolic NE81 assemblies. This method became available when we found that GFP-NE81 with a non-functional nuclear localization sequence and lacking a CaaX-box (GFP-NE81ΔNLSΔCLIM with replacement of all basic residues of the canonical NLS by alanine residues) formed cell cycle-dependent green fluorescent clusters in the cytosol (Figure 6A). GFP-NE81ΔNLSΔCLIM clusters dissolved at the onset of mitosis and re-formed in telophase (Figure 6A, supplemental movie 3). This behavior corresponded exactly to that of the respective intranuclear clusters of GFP-NE81ΔCLIM with a functional NLS [18]. In our previous work we presented data indicating that these clusters represent protein assemblies regulated by CDK1, which initiates their disassembly at mitotic onset. GFP-NE81ΔNLSΔCLIM clusters not only show the same assembly/disassembly behavior, they also exhibited the same spongy appearance in electron microscopy images of ultrathin slices as the intranuclear GFP-NE81ΔNLSΔCLIM clusters published previously (Figure 6B,C) [18].

In animal cells, binding of importin α to the NLS of nascent prelamin prevents premature cytosolic assembly of freshly translated lamin prior to its import into the nucleus [43]. Provided that a similar situation holds true for *Dictyostelium*, the observation that cytosolic GFP-NE81ΔNLSΔCLIM clusters were studded with ribosomes suggests that they assemble co-translationally, likely because the lack of a functional NLS prevents importin α from inhibiting assembly of nascent NE81 polypeptides as soon as their coiled coil regions are translated (Figure 6B,C). In this work we used these cytosolic GFP-NE81ΔNLSΔCLIM clusters to investigate whether they were able to bind Src1 fragments corresponding to nucleoplasmic portions of the full length protein. We chose two soluble fragments, one preceding the first transmembrane domain (aa 356–565) and the second one comprising the whole C-terminus after the second transmembrane domain (aa 826–943) (Figure 6D). Both fragments were devoid of strong nuclear localization sequences predicted with NLS mapper [44]. When these fragments were expressed as red-fluorescent mRFP-fusion proteins in *Dictyostelium* AX2 control cells, both fragments, mRFP-Src1^356–565^ and mRFP-Src1^826–943^, showed an even distribution within the cytosol with some accumulation in the nucleus, especially in case of mRFP-Src1^826–943^ (Figure 6E,F). However, if expressed in GFP-NE81ΔNLSΔCLIM cells, both red-fluorescent fragments also strongly localized to the cytosolic green-fluorescent GFP-NE81ΔNLSΔCLIM clusters (Figure 6G,H). This clearly indicates binding of Src1 to NE81. Taken together, we have two lines of evidence that Src1 contributes to the organization or architecture of the nuclear lamina through an interaction with the lamin NE81. However, we cannot exclude an indirect interaction between both proteins (see discussion).

**Figure 7:**
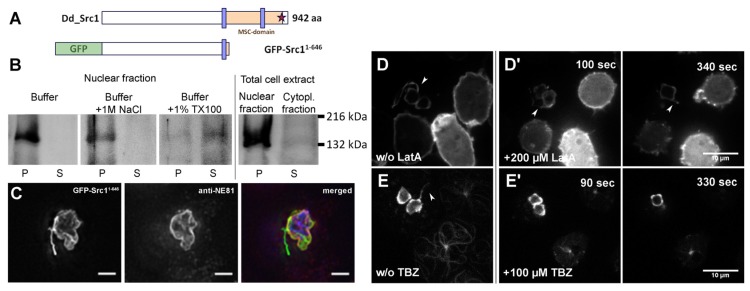
Nuclear envelope protrusions caused by expression of GFP-Src1^1–646^ are dependent on intact microtubules but not actin filaments. (**A**) Schematic of GFP-Src1^1–646^. (**B**) Western Blot stained with anti-GFP antibodies showing GFP-Src1^1–646^ membrane association after extractions with buffer (control), high-salt, and detergent, respectively. GFP-Src1^1–646^ is a nuclear membrane protein, since it becomes solubilized only by extraction with 1% Triton-X100. (**C**) Immunofluorescence microscopy of paraformaldehyde fixed GFP-Src1^1–646^ (green) cells stained with anti-NE81/AlexaFluor 568 (red) and DAPI (blue). Bar, 5 μm. Note that the origin of the nuclear envelope protrusion (arrowhead) is associated with a nucleolus. (**D**–**E**) Selected time points of supplemental movie 4 (**D**) and movie 5 (**E**) showing the dynamic behavior of nuclear membrane protrusions (arrowhead). GFP-Src1^1–646^ cells (hash tag) were mixed 1:1 either with GFP-LIMΔcoil cells(asterisk) [45] with green fluorescent F-actin (**D**) or with GFP-α-tubulin cells (asterisk) [46] with green fluorescent microtubules (**E**) to monitor effectiveness of drug treatments. The presence of protusions was not affected by treatment with 200 μM latrunculin A (LatA, **D'**) or 100 μM thiabendazole (TBZ, **E'**). Cells were viewed under agar overlay. Bars, 10 μm.

The interaction of Src1 with the nuclear lamina protein NE81 suggested that Src1 could also be required for integrity of the nuclear envelope. This idea was supported by the overexpression phenotype observed upon expression of an Src1 fragment comprising the N-terminal half including the first transmembrane domain (aa 1–646) as a GFP-fusion protein (GFP-Src^1–646^; Figure 7A). We could not estimate the extent of its overexpression in Western blotted cell extracts directly, since our anti-Src1 antibody was raised against a part of the protein missing in GFP-Src^1–646^. However, when we stained extracts from GFP-Src1 (full length) and GFP-Src^1–646^ cells with both anti-Src1 and anti-GFP antibodies we could estimate relative staining intensities of the respective proteins (Figure 2C). According to this estimation GFP-Src^1–646^ is overexpressed more than 7-fold. Protein extraction experiments confirmed anchoring of this fragment at nuclear membranes, since it could be extracted from the membrane fraction by detergent extraction with Triton-X100, but not by high-salt treatment (Figure 7B). GFP-Src^1–646^ cells were characterized by protrusions of the nuclear envelope. Protrusions were associated with NE81 at most at their origin but not through their length, indicating that association of the nuclear envelope with the nuclear lamina is compromised in these cells (Figure 7C). Many protrusions showed a striking dynamic behavior *i.e.*, they popped up at various locations at the nuclear envelope, growing, shrinking, and disappearing arbitrarily (Figure 7D,E; supplemental movie 4 and 5). This behavior was widely unaffected by treatment of cells with the actin depolymerizing drug latrunculin A (latA), but clearly diminished upon incubation with the microtubule-depolymerizing drug thiabendazole (TBZ) (Figure 7D,E; supplemental movie 4 and 5). Thus, the observed nuclear protrusions resulted from mechanical forces transmitted through the microtubule cytoskeleton with associated motor proteins.

## 4. Discussion

In this paper we provide strong evidence that Src1, the only HeH-family member in *Dictyostelium discoideum*, interacts with the *Dictyostelium* lamin NE81 and participates in formation of the nuclear lamina. Although we cannot exclude an indirect interaction between both proteins, a direct interaction is supported by their close proximity detected by BioID and by the fact that our mis-localization assay targets both normally nuclear proteins to the cytosol where natural interaction partners that could mediate an indirect interaction are not expected in sufficient amounts. The nuclear lamina of this organism is not thoroughly characterized, however there is no doubt that NE81 requires farnesylation for proper assembly at the INM and it is conceivable that it behaves like a B-type lamin, *i.e.*, that it retains its farnesyl anchor throughout the cell cycle [18]. As in other organisms the lamin network may be connected through both the farnesyl anchor and also through interactions with inner nuclear membrane proteins. A role of Src1 in maintenance of nuclear envelope integrity became evident by the dominant-negative effect of expression of the GFP-Src^1–646^ fragment. This fusion protein lacks one of the two identified NE81 interaction domains and should have reduced affinity to NE81 assemblies beneath the INM. As this fragment still contains one transmembrane domain and the predicted NLSs, it could replace endogenous Src1 to some extent at the INM and compromise association of NE81 networks to the INM. According to our indirect densitometric blot evaluation GFP-Src^1–646^ is overexpressed more than seven-fold. This is in agreement with the strong phenotype of GFP-Src^1–646^ cells, in which dynamic nuclear envelope protrusions indicated that the nuclear envelope was no longer capable of withstanding the pulling and pushing forces exerted by the microtubule cytoskeleton with its associated motors. As in many animal cells, in *Dictyostelium* microtubules emanating from the nucleus-associated centrosome are nestling all around the nucleus. Due to the fact that dynein is also localized at the nucleus [47], it is conceivable that microtubules in the nuclear vicinity interact with dynein at the nuclear surface. The nuclear protrusion phenotype of GFP-Src^1–646^ cells clearly supports the idea that the GFP-fusion protein is unable to interact properly with NE81 at the nuclear lamina, thus allowing formation of nuclear protrusions, whereby motor forces exerted by dynein contribute to their dynamic behavior. Interestingly, nuclear protrusions occured at sites associated with nucleoli (Figure 7D) and, thus, they were reminiscent of so-called “nuclear nozzles” described in aggregating (but not vegetative) *Dictyostelium* cells by Sameshima and co-workers [48,49]. In these polarized developing cells a long extension of the nuclear envelope associated with a nucleolus was typically observed at the anterior end of the nucleus. The significance of this structure is still unknown, however, its presence was dependent of intact microtubules as it disappeared upon treatment with nocodazole [49].

In contrast to NE81, Src1 was not evenly distributed around the nuclear envelope, but concentrated at sites of nucleolar attachment to the nuclear envelope. This suggests that Src1 is not only required for stability of the nuclear envelope, but that it may also be involved in functions of the nucleolus. This would not be without precedent. As mentioned in the introduction, budding yeast Src1 is involved in stabilization of highly repetitive rDNA sequences at the nuclear periphery in cooperation with other proteins [12], and the cell-cycle regulated *Schizosaccharomyces japonicus* Man1 appears to be required for nucleolar disassembly by regulating condensation of rDNA arrays [13]. In *Dictyostelium*, cell cycle-dependent regulation of *src1* is also likely, according to observations by Prof. Harry K. MacWilliams (†) (LMU Munich, March 2010, [50]; otherwise unpublished). In this context *src1* might promote compaction of rDNA and dissolution of nucleoli that typically takes place at this cell cycle stage [51]. This could also involve phosphorylation of the predicted consensus sequence for CDK1 phosphorylation near the C-terminus of Src1. Future analyses will reveal whether the predicted CDK1 consensus sequence close to the C-terminal end of Src1 could be involved in this process. Furthermore, it remains to be seen how the Src1-NE81-interaction is involved in nucleolar organization and whether this interaction could also be involved in other Src1 functions, such as anchorage of subtelomeric chromatin, another known function of fungal HeH-proteins [52,53,54]. 

HeH-proteins along with Sun-proteins and lamins most likely were present already in the last common ancestor of eukaryotes (LECA) [16,20,21,22]. Among all unicellular model organisms *Dictyostelium* is the only one, in which orthologues of all three mentioned protein families are expressed and, thus, may most closely reflect the situation in LECA. This makes it a very promising system to investigate the minimal requirements of nuclear lamina organization and the basic roles of the interplay between INM proteins (such as Src1, Sun1, and lamins) and chromatin.

## Supplementary Materials

The following are available online at www.mdpi.com/2073-4409/5/1/13/s1, **Movie 1.** Localization of GFP-Src1 during interphase and mitosis. Maximum intensity projection of image stacks consisting of seven frames (step size 0.270 μm) recorded every 10 s at a frame rate of 10 fr/s. **Movie 2.** Mobility of GFP-Src1 in FRAP experiments. The bleached region is indicated by a white square. Maximum intensity projection of image stacks consisting of seven frames (step size 0.270 μm) recorded every 10 s at a frame rate of 10 fr/s. **Movie 3.** Dynamic behavior of GFP-NE81ΔNLSΔCLIM clusters. Recording from late G2 till mitotic exit. Maximum intensity projection of image stacks consisting of three frames (step size 1 μm) recorded every 10 s at a frame rate of 10 fr/s. **Movie 4.** Dynamic behavior of nuclear membrane protrusions in GFP-Src1^1–646^ cells is unaffected by treatment with LatA. Cells were mixed 1:1 with GFP-LIMΔcoil cells with green fluorescent F-actin to monitor effectiveness of drug treatment. The time point of TBZ addition is indicated. Cells were viewed under agar overlay. Maximum intensity projection of image stacks consisting of five frames (step size 0.270 μm) recorded every 20 s at a frame rate of 2 fr/s. **Movie 5.** Dynamic behavior of nuclear membrane protrusions in GFP-Src1^1–646^ cells is compromised by treatment with TBZ. Cells were mixed 1:1 with GFP-α-tubulin cells with green fluorescent microtubules to monitor effectiveness of drug treatment. The time point of TBZ addition is indicated. Cells were viewed under agar overlay. Maximum intensity projection of image stacks consisting of three frames (step size 0.270 μm) recorded every 10 s at a frame rate of 2 fr/s.

## Abbreviations

The following abbreviations are used in this manuscript:

BioID: Biotin identification
HeH: Helix-extension-helix
NE: Nuclear envelope
INM: Inner nuclear membrane
ONM: Outer nuclear membrane
